# Supplementary material for: Disturbed Plasma Lipidomic Profiles in Females with Diffuse Large B-Cell Lymphoma: A Pilot Study
Source: Cancers (Basel). 2023 Jul 18;15(14):3653. doi: 10.3390/cancers15143653 (PMC10377844; doi:10.3390/cancers15143653)
Supplement: Supplementary file 1 [file cancers-15-03653-s001.zip › Masnikosa et al Supplementary File S1.pdf]

SUPPLEMENTARY FILE 1

For the manuscript:

DISTURBED PLASMA LIPIDOMIC PROFILES IN FEMALES WITH DIFFUSE LARGE B-CELL LYMPHOMA: A PILOT STUDY

Romana Masnikosa[1]*, David Pirić[1], Julia Maria Post[2], Zorica Cvetković[3,4], Snježana Petrović[5], Marija Paunović[5], Vesna Vučić[5], Laura Bindila[2*]

1 Department of Physical Chemistry, Vinca Institute of Nuclear Sciences - National Institute of the Republic of Serbia, University of Belgrade, Mike Petrovica Alasa 12-14, 11001 Belgrade, Republic of Serbia

2 Clinical Lipidomics Unit, Institute of Physiological Chemistry, University Medical Center, Duesbergweg 6, 55128 Mainz, Germany

3 Department of Haematology, Clinical Hospital Center Zemun, Vukova 9, 11080 Belgrade, Republic of Serbia

4 Faculty of Medicine, University of Belgrade, Dr Subotica 8, 11000 Belgrade, Republic of Serbia

5 Group for Nutritional Biochemistry and Dietology, Centre of Research Excellence in Nutrition and Metabolism, Institute for Medical Research, National Institute of the Republic of Serbia, University of Belgrade, Tadeusa Koscuska 1, 11000 Belgrade, Republic of Serbia

*Corresponding authors: Dr Laura Bindila. email: bindila@uni-mainz.de phone: +4961313925794, fax: +4961313923536, and dr Romana Masnikosa, e-mail: romana@vin.bg.ac.rs, phone +38162436343

Table S1. Plasma lipid species measured by the untargeted lipidomics (semi-quantification)

Abbreviation	Lipid species	Mean conc. (nmol/mL)
PC 14:0_18:2	1-tetradecanoyl-2-octadecadienoyl-sn-glycero-3-phosphocholine	6.830
PC 14:0_20:4	1-tetradecanoyl-2-eicosatetraenoyl-sn-glycero-3-phosphocholine	2.392
PC 14:0_22:6	1-tetradecanoyl-2-docosahexaenoyl-sn-glycero-3-phosphocholine	0.851
PC 15:0_16:0	1-pentadecanoyl-2-hexadecanoyl-sn-glycero-3-phosphocholine	1.264
PC 15:0_20:4	1-pentadecanoyl-2- eicosatetraenoyl-sn-glycero-3-phosphocholine	1.892
PC 16:0_14:0	1-hexadecanoyl-2-tetradecanoyl-sn-glycero-3-phosphocholine	4.429
PC 16:0_16:0	1-hexadecanoyl-2-hexadecanoyl-sn-glycero-3-phosphocholine	26.513
PC 16:0_18:1	1-hexadecanoyl-2-octadecenoyl-sn-glycero-3-phosphocholine	283.179
PC 16:0_18:2	1-hexadecanoyl-2-octadecadienoyl-sn-glycero-3-phosphocholine	555.483
PC 16:0_20:4	1-hexadecanoyl-2-eicosatetraenoyl-sn-glycero-3-phosphocholine	223.120
PC 16:0_20:5	1-hexadecanoyl-2-eicosapentaenoyl-sn-glycero-3-phosphocholine	15.352
PC 16:0_22:6	1-hexadecanoyl-2-docosahexaenoyl-sn-glycero-3-phosphocholine	75.234
PC 16:1_18:2	1-hexadecenoyl-2-octadecadienoyl-sn-glycero-3-phosphocholine	12.248
PC 16:1_22:6	1-hexadecenoyl-2-docosahexaenoyl-sn-glycero-3-phosphocholine	0.685
PC 17:0_14:1	1-heptadecanoyl-2-tetradecenoyl-sn-glycero-3-phosphocholine	0.214
PC 17:0_18:1	1-heptadecanoyl-2-octadecenoyl-sn-glycero-3-phosphocholine	4.079
PC 17:0_18:2	1-heptadecanoyl-2-octadecadienoyl-sn-glycero-3-phosphocholine	11.747
PC 17:0_20:4	1-heptadecanoyl-2-eicosatetraenoyl-sn-glycero-3-phosphocholine	5.828
PC 17:0_22:6	1-heptadecanoyl-2-docosahexaenoyl-sn-glycero-3-phosphocholine	1.630
PC 17:1_18:2	1-heptadecenoyl-2-octadecadienoyl-sn-glycero-3-phosphocholine	1.593
PC 17:1_20:4	1-heptadecenoyl-2-eicosatetraenoyl-sn-glycero-3-phosphocholine	0.698
PC 18:0_16:0	1-octadecanoyl-2-hexadecanoyl-sn-glycero-3-phosphocholine	4.285
PC 18:0_18:1	1-octadecanoyl-2-octadecenoyl-sn-glycero-3-phosphocholine	50.685
PC 18:0_18:2	1-octadecanoyl-2-octadecadienoyl-sn-glycero-3-phosphocholine	366.417

PC 18:0_20:2	1-octadecanoyl-2-eicosadienoyl-sn-glycero-3-phosphocholine	7.034
PC 18:0_20:4	1-octadecanoyl-2-eicosatetraenoyl-sn-glycero-3-phosphocholine	151.117
PC 18:0_22:4	1-octadecanoyl-2-docosatetraenoyl-sn-glycero-3-phosphocholine	5.842
PC 18:0_22:5	1-octadecanoyl-2-docosapentaenoyl-sn-glycero-3-phosphocholine	4.356
PC 18:0_22:6	1-octadecanoyl-2-docosahexaenoyl-sn-glycero-3-phosphocholine	32.360
PC 18:1_18:2	1-hexadecenoyl-2-octadecadienoyl-sn-glycero-3-phosphocholine	153.268
PC 18:1_20:4	1-hexadecenoyl-2-eicosatetraenoyl-sn-glycero-3-phosphocholine	40.709
PC 18:2_18:2	1-octadecadienoyl-2-octadecadienoyl-sn-glycero-3-phosphocholine	33.617
PC 18:2_20:4	1-octadecadienoyl-2-eicosatetraenoyl-sn-glycero-3-phosphocholine	11.325
PC 19:0_18:2	1-nonadecanoyl-2-octadecadienoyl-sn-glycero-3-phosphocholine	2.010
PC 20:1_18:2	1-eicosenoyl-2-octadecadienoyl-sn-glycero-3-phosphocholine	43.740
PC 20:2_20:4	1-eicosadienoyl-2-eicosatetraenoyl-sn-glycero-3-phosphocholine	2.305
PC 20:4_20:4	1-eicosatetraenoyl-2-eicosatetraenoyl-sn-glycero-3-phosphocholine	1.260
PC O-18:1_20:4	1-octadecenyl-2-eicosatetraenoyl-sn-glycero-3-phosphocholine	0.232
PC O-20:1_20:4	1-eicosenyl-2-eicosatetraenoyl-sn-glycero-3-phosphocholine	0.274
LPC 14:0	tetradecanoyl-sn-glycero-3-phosphocholine	0.526
LPC 15:0 sn-1	1-pentadecanoyl-sn-glycero-3-phosphocholine	0.287
LPC 16:0 sn-1	1-hexadecanoyl-sn-glycero-3-phosphocholine	47.545
LPC 18:0 sn-1	1-octadecanoyl-sn-glycero-3-phosphocholine	21.091
LPC 18:1 sn-1	1-octadecenoyl-sn-glycero-3-phosphocholine	10.672
LPC 18:2 sn-1	1-octadecadienoyl-sn-glycero-3-phosphocholine	17.625
LPC 18:3	octadecatrienoyl-sn-glycero-3-phosphocholine	0.169
LPC 19:0	nonadecanoyl-sn-glycero-3-phosphocholine	0.107
LPC 20:4 sn-1	1-eicosatetraenoyl-sn-glycero-3-phosphocholine	3.834
LPC 20:4 sn-2	2-eicosatetraenoyl-sn-glycero-3-phosphocholine	1.042
LPC 20:5	eicosapentaenoyl-sn-glycero-3-phosphocholine	0.206
LPC 22:5 sn-1	1-docosapentaenoyl-sn-glycero-3-phosphocholine	0.177
LPC 22:6 sn-1	1-docosahexaenoyl-sn-glycero-3-phosphocholine	0.761
LPC 22:6 sn-2	2-docosahexaenoyl-sn-glycero-3-phosphocholine	0.226
LPC 24:0	tetracosanoyl-sn-glycero-3-phosphocholine	0.090
SM 34:0;O3		0.421
SM 34:1;O3	N-(2-hydroxyhexadecanoyl)-sphing-4-enine-1-phosphocholine	0.978
SM d18:1_16:0	N-(hexadecanoyl)-sphing-4-enine-1-phosphocholine	130.852
SM d18:1_16:1	N-(hexadecenoyl)-sphing-4-enine-1-phosphocholine	23.672
SM d18:1_18:0	N-(octadecanoyl)-sphing-4-enine-1-phosphocholine	27.574
SM d18:1_18:1	N-(octadecenoyl)-sphing-4-enine-1-phosphocholine	15.976

SM d18:1_24:0	N-(tetracosanoyl)-sphing-4-enine-1-phosphocholine	29.010
SM d18:1_24:1	N-(tetracosenoyl)-sphing-4-enine-1-phosphocholine	55.995
SM d24:2_14:0	N-(tetradecanoyl)-tetracosasphingadienine-1-phosphocholine	8.672
SM d32:1	Sphingomyelin d32:1	15.130
SM d32:2	Sphingomyelin d32:2	1.341
SM d33:1	Sphingomyelin d33:1	9.186
SM d34:0	Sphingomyelin d34:0	6.949
SM d35:1	Sphingomyelin d35:1	1.023
SM d35:2	Sphingomyelin d35:2	0.751
SM d36:3	Sphingomyelin d36:3	0.802
SM d38:1	Sphingomyelin d38:1	18.346
SM d38:3	Sphingomyelin d38:3	0.488
SM d39:1	Sphingomyelin d39:1	7.497
SM d40:1	Sphingomyelin d40:1	45.635
SM d40:2	Sphingomyelin d40:2	5.128
SM d40:3	Sphingomyelin d40:3	2.183
SM d41:1	Sphingomyelin d41:1	19.162
SM d41:2	Sphingomyelin d41:2	0.638
SM d41:3	Sphingomyelin d41:3	1.270
SM d44:2	Sphingomyelin d44:2	0.419
SM d44:3	Sphingomyelin d44:3	0.595
Cer 18:2;O2/24:1	Ceramide 18:2;O2/24:1	1.968
Cer d18:1_16:0	N-(hexadecanoyl)-sphing-4-enine	1.834
Cer d18:1_20:0	N-(eicosanoyl)-sphing-4-enine	1.143
Cer d18:1_22:0	N-(docosanoyl)-sphing-4-enine	5.807
Cer d18:1_24:0	N-(tetracosanoyl)-sphing-4-enine	18.089
Cer d18:1_24:1	N-(tetracosenoyl)-sphing-4-enine	7.072
Cer d40:2	Ceramide d40:2	1.475
Cer d41:1	Ceramide d41:1	4.846
Cer d43:1	Ceramide d43:1	3.065
Cer d43:2	Ceramide d43:2	0.606
Cer d44:1	Ceramide d44:1	0.806
PE 16:0_18:1	1-hexadecanoyl-2-octadecenoyl-sn-glycero-3-phosphoethanolamine	0.995
PE 16:0_18:2	1-hexadecanoyl-2-octadecadienoyl-sn-glycero-3-phosphoethanolamine	1.983
PE 16:0_20:4	1-hexadecanoyl-2-eicosatetraenoyl-sn-glycero-3-phosphoethanolamine	2.708
PE 16:0_22:6	1-hexadecanoyl-2-docosahexaenoyl-sn-glycero-3-phosphoethanolamine	2.790

PE 18:0_18:1	1-octadecanoyl-2-octadecenoyl-sn-glycero-3-phosphoethanolamine	2.767
PE 18:0_18:2	1-octadecanoyl-2-octadecadienoyl-sn-glycero-3-phosphoethanolamine	4.104
PE 18:0_20:3	1-octadecanoyl-2-eicosatrienoyl-sn-glycero-3-phosphoethanolamine	0.443
PE 18:0_20:4	1-octadecanoyl-2-eicosatetraenoyl-sn-glycero-3-phosphoethanolamine	7.051
PE 18:0_22:6	1-octadecanoyl-2-docosahexaenoyl-sn-glycero-3-phosphoethanolamine	1.731
PE 18:1_18:2	1-octadecenoyl-2-octadecadienoyl-sn-glycero-3-phosphoethanolamine	1.002
PE 18:1_20:4	1-octadecenoyl-2-eicosatetraenoyl-sn-glycero-3-phosphoethanolamine	1.118
PE 18:2_18:2	1-octadecenoyl-2-octadecenoyl-sn-glycero-3-phosphoethanolamine	0.178
PE 18:2_20:4	1-octadecenoyl-2-eicosatetraenoyl-sn-glycero-3-phosphoethanolamine	0.164
PE O-16:1_18:1	1-hexadecenyl-2-octadecenoyl-sn-glycero-3-phosphoethanolamine	0.779
PE O-16:1_18:2	1-hexadecenyl-2-octadecadienoyl-sn-glycero-3-phosphoethanolamine	1.577
PE O-16:1_20:4	eicosatetraenoyl-sn-glycero-3-phosphoethanolamine	5.236
PE O-16:1_22:6	1-hexadecenyl-2-docosahexaenoyl-sn-glycero-3-phosphoethanolamine	2.577
PE O-17:1_18:2	1-heptadecenyl-2-octadecadienoyl-sn-glycero-3-phosphoethanolamine	0.200
PE O-17:1_20:4	1-heptadecenyl-2-eicosatetraenoyl-sn-glycero-3-phosphoethanolamine	0.462
PE O-17:1_22:6	1-heptadecenyl-2-docosahexaenoyl-sn-glycero-3-phosphoethanolamine	0.201
PE O-18:1_20:1	1-octadecenyl-2-eicosenoyl-sn-glycero-3-phosphoethanolamine	0.168
PE O-18:1_20:3	1-octadecenyl-2-eicosatrienoyl-sn-glycero-3-phosphoethanolamine	0.671
PE O-18:1_20:5	1-octadecenyl-2-eicosapentaenoyl-sn-glycero-3-phosphoethanolamine	1.133
PE O-18:1_22:4	1-octadecenyl-2-docosatetraenoyl-sn-glycero-3-phosphoethanolamine	0.370
PE O-18:2_20:4	1-octadecadienyl-2-eicosatetraenoyl-sn-glycero-3-phosphoethanolamine	3.895
PE O-18:2_22:6	1-octadecadienyl-2-docosahexaenoyl-sn-glycero-3-phosphoethanolamine	1.534
PE O-18:3_20:4	1-octadecatrienyl-2-eicosatetraenoyl-sn-glycero-3-phosphoethanolamine	0.413
PE O-20:1_18:2	1-eicosenyl-2-octadecadienoyl-sn-glycero-3-phosphoethanolamine	0.367
PE O-20:1_20:4	1-eicosenyl-2-eicosatetraenoyl-sn-glycero-3-phosphoethanolamine	0.768
PE O-20:2_22:6	1-eicosadienyl-2-docosahexaenoyl-sn-glycero-3-phosphoethanolamine	0.133
PE O-22:1_20:4	1-docosenyl-2-eicosatetraenoyl-sn-glycero-3-phosphoethanolamine	0.148
PE O-22:2_20:4	1-docosadienyl-2-eicosatetraenoyl-sn-glycero-3-phosphoethanolamine	0.259
PE O-24:2_20:4	1-tetracosadienyl-2-eicosatetraenoyl-sn-glycero-3-phosphoethanolamine	0.195
LPE 16:0 sn-1	1-hexadecanoyl-sn-glycero-3-phosphoethanolamine	2.950
LPE 16:0 sn-2	2-hexadecanoyl-sn-glycero-3-phosphoethanolamine	0.687
LPE 18:0 sn-1	1-octadecanoyl-sn-glycero-3-phosphoethanolamine	3.790
LPE 18:0 sn-2	2-octadecanoyl-sn-glycero-3-phosphoethanolamine	0.578
LPE 18:1 sn-1	1-octadecenoyl-sn-glycero-3-phosphoethanolamine	2.512
LPE 18:2 sn-1	1-octadecadienoyl-sn-glycero-3-phosphoethanolamine	5.397
LPE 18:2 sn-2	2-octadecadienoyl-sn-glycero-3-phosphoethanolamine	1.322

LPE 20:4 sn-1	1-eicosatetraenoyl-sn-glycero-3-phosphoethanolamine	2.213
LPE 20:4 sn-2	2-eicosatetraenoyl-sn-glycero-3-phosphoethanolamine	4.294
LPE 22:5	docosapentaenoyl-sn-glycero-3-phosphoethanolamine	0.377
LPE 22:6 sn-1	1-docosahexaenoyl-sn-glycero-3-phosphoethanolamine	2.673
LPE 22:6 sn-2	2-docosahexaenoyl-sn-glycero-3-phosphoethanolamine	1.223
LPE O-18:1	octadecenyl-sn-glycero-3-phosphoethanolamine	0.539
PI 16:0_16:0	1-hexadecanoyl-2-hexadecanoyl-sn-glycero-3-phosphoinositol	0.315
PI 16:0_16:1	1-hexadecanoyl-2-hexadecenoyl-sn-glycero-3-phosphoinositol	0.417
PI 16:0_18:1	1-hexadecanoyl-2-octadecenoyl-sn-glycero-3-phosphoinositol	2.018
PI 16:0_18:2	1-hexadecanoyl-2-octadecadienoyl-sn-glycero-3-phosphoinositol	3.821
PI 16:0_20:4	1-hexadecanoyl-2-eicosatetraenoyl-sn-glycero-3-phosphoinositol	3.556
PI 18:0_18:1	1-octadecanoyl-2-octadecenoyl-sn-glycero-3-phosphoinositol	1.811
PI 18:0_18:2	1-octadecanoyl-2-octadecadienoyl-sn-glycero-3-phosphoinositol	8.838
PI 18:0_20:3	1-octadecanoyl-2-eicosatrienoyl-sn-glycero-3-phosphoinositol	2.108
PI 18:0_20:4	1-octadecanoyl-2-eicosatetraenoyl-sn-glycero-3-phosphoinositol	28.602
PI 18:0_22:4	1-octadecanoyl-2-docosatetraenoyl-sn-glycero-3-phosphoinositol	0.285
PI 18:0_22:5	1-octadecanoyl-2-docosapentaenoyl-sn-glycero-3-phosphoinositol	0.583
PI 18:0_22:6	1-octadecanoyl-2-docosahexaenoyl-sn-glycero-3-phosphoinositol	1.036
PI 18:1_18:2	1-octadecenoyl-2-octadecadienoyl-sn-glycero-3-phosphoinositol	1.405
PI 18:1_20:4	1-octadecenoyl-2-eicosatetraenoyl-sn-glycero-3-phosphoinositol	0.955
PS 18:0_18:0	1-octadecanoyl-2-octadecanoyl-sn-glycero-3-phosphoserine	0.955
PS 18:0_18:1	1-octadecanoyl-2-octadecenoyl-sn-glycero-3-phosphoserine	1.464
PS 18:0_20:4	1-octadecanoyl-2-eicosatetraenoyl-sn-glycero-3-phosphoserine	0.099

Lipid species were measured in plasma samples of diffuse large B-cell lymphoma (DLBCL) patients and controls individuals (CTRL). Mean concentration (mean conc.) for all analytes was calculated (17 DLBCL + 21CTRL).

Table S2. The summary of the principal component analysis (PCA) of the lipid dataset obtained through the untargeted lipidomics (38 x155)

**Principal component**	**PC1**	**PC2**	**PC3**	**PC4**	**PC5**	**PC6**	**PC7**
Eigenvalue	42.99	16.35	12.62	10.28	8.78	7.59	6.80
Proportion of variance (%)	27.74	10.55	8.15	6.63	5.66	4.89	4.39
Cumulative proportion of variance (%)	27.74	38.28	46.43	53.06	58.72	63.61	68.00

Table S3. Summary of OPLS-DA model built from the lipid dataset obtained through the untargeted lipidomics (38 x 155)

OPLS-DA model	Model characteristics
samples	38
X variables	155
excluded_X_variables	0
Y_variables	1
missing_values	0 (0%)
R2X(cum)	0.362
R2Y(cum)	0.758
Q2(cum)	0.513
RMSEE	0.255
# correctly classified DLBCL	16/17
# correctly classified CTRL	21/21
Fischer' s probability	7.6×10^{-10}
CV-ANOVA of the model	
F-value	8.685
SD	2.178
p-value	6.64×10^{-5}

Table S4. VIP values from the OPLS-DA model built from the 38 x 155 lipid dataset (lipid species with VIP > 1 are shown)

Lipid species	VIP value
PC 18:2_18:2	2.250
PI 18:1_18:2	2.044
PE 18:0_18:1	2.010
PC 18:0_18:2	1.977
PC 18:2_20:4	1.936
LPE 18:2 sn-2	1.929
PE O-16:1_18:1	1.905
PE O-16:1_18:2	1.904
LPC 18:2 sn-1	1.838
Cer d18:1_24:1	1.818
Cer d44:1	1.808
PI 18:0_18:1	1.799
PS 18:0_18:1	1.734
PE O-17:1_18:2	1.664
PC 14:0_18:2	1.636
PI 18:1_20:4	1.626
PE O-20:1_18:2	1.616
PC 16:1_18:2	1.610
PC O-18:1_20:4	1.600
LPE 18:2 sn-1	1.566
SM d39:1	1.536
PC 18:1_18:2	1.512
PC 17:0_18:2	1.500
PC 16:0_18:2	1.470
SM d38:1	1.428
PI 16:0_18:1	1.409
SM d32:2	1.408
SM d32:1	1.382
PC 17:1_18:2	1.367
PI 16:0_18:2	1.346
Cer d40:2	1.338
PC 14:0_20:4	1.330
SM 34:0;3O	1.314
SM d44:2	1.303
PE O-18:3_20:4	1.251
PI 18:0_18:2	1.247
PE O-18:2_20:4	1.246
PC 20:4_20:4	1.242
PE O-17:1_20:4	1.224
PE O-16:1_20:4	1.221

LPE 18:1 sn-1	1.207
PC 18:0_18:1	1.204
LPE 22:5	1.184
PE 18:0_22:6	1.178
Cer d18:1_16:0	1.176
Cer d43:2	1.176
SM d33:1	1.154
SM d18:1_18:0	1.142
PE 16:0_20:4	1.120
SM d18:1_24:1	1.115
PI 18:0_20:3	1.098
PC 19:0_18:2	1.083
PS 18:0_20:4	1.083
LPC 19:0	1.024
PE 18:0_20:4	1.003
PE O-17:1_22:6	1.001

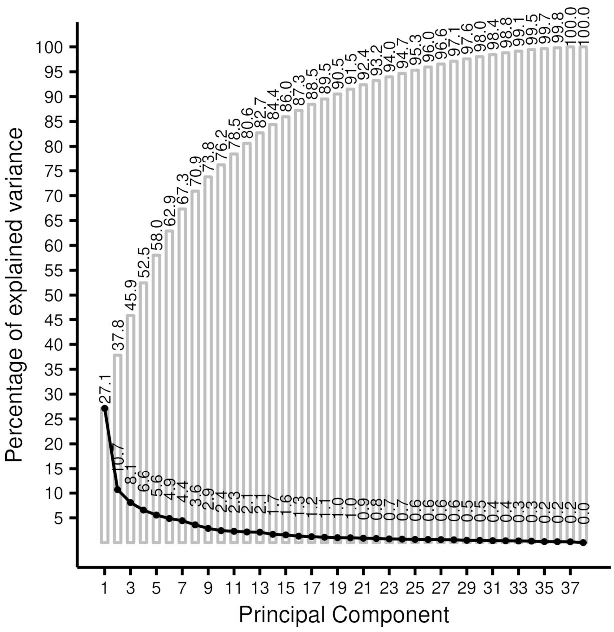

Figure S1. Scree plot. Proportion of variance explained by the principal components (black line: individual; grey columns: cumulative variance).
