# Supplementary material for: Disturbed Plasma Lipidomic Profiles in Females with Diffuse Large B-Cell Lymphoma: A Pilot Study
Source: Cancers (Basel). 2023 Jul 18;15(14):3653. doi: 10.3390/cancers15143653 (PMC10377844; doi:10.3390/cancers15143653)
Supplement: Supplementary file 1 [file cancers-15-03653-s001.zip › Masnikosa et al Supplementary File S2.pdf]

SUPPLEMENTARY FILE 2

For the manuscript:

DISTURBED PLASMA LIPIDOMIC PROFILES IN FEMALES WITH DIFFUSE LARGE B-CELL LYMPHOMA: A PILOT STUDY

Romana Masnikosa[1]*, David Pirić[1], Julia Maria Post[2], Zorica Cvetković[3,4], Snježana Petrović[5], Marija Paunović[5], Vesna Vučić[5], Laura Bindila[2*]

1 Department of Physical Chemistry, Vinca Institute of Nuclear Sciences – National Institute of the Republic of Serbia, University of Belgrade, Mike Petrovica Alasa 12-14, 11001 Belgrade, Republic of Serbia

2 Clinical Lipidomics Unit, Institute of Physiological Chemistry, University Medical Center, Duesbergweg 6, 55128 Mainz, Germany

3 Department of Haematology, Clinical Hospital Center Zemun, Vukova 9, 11080 Belgrade, Republic of Serbia

4 Faculty of Medicine, University of Belgrade, Dr Subotića 8, 11000 Belgrade, Republic of Serbia

5 Group for Nutritional Biochemistry and Dietology, Centre of Research Excellence in Nutrition and Metabolism, Institute for Medical Research, National Institute of the Republic of Serbia, University of Belgrade, Tadeusa Koscuska 1, 11000 Belgrade, Republic of Serbia

*Corresponding authors: Dr Laura Bindila. email: bindila@uni-mainz.de phone: +4961313925794, fax: +4961313923536, and dr Romana Masnikosa, e-mail: romana@vin.bg.ac.rs, phone +38162436343

Table S1. Calibration standards used for the quantification of 35 plasma lipid species by LC-MRM

	abbreviation	lipid species	Target conc. range (ng/mL)
1	AA	arachidonic acid FA 20:4	160 – 6400
2	TxB2	thromboxane B2	0.2 - 8
3	5(S)-HETE	5S-hidroxyeicosatetraenoic acid	0.4 - 16
4	8(S)-HETE	8S-hidroxyeicosatetraenoic acid	0.4 - 16
5	12(S)-HETE	12S-hidroxyeicosatetraenoic acid	0.4 - 16
6	15(S)-HETE	15S-hidroxyeicosatetraenoic acid	0.4 - 16
7	20-HETE	20-hidroxyeicosatetraenoic acid	0.2 - 8
8	5(6)-DiHET	5,6-dihidroxy-8Z,11Z,14Z-eicosatrienoic acid	0.4 - 16
9	8(9)-DiHET	8,9-dihidroxy-5Z,11Z,14Z-eicosatrienoic acid	0.4 - 16
10	11(12)-DiHET	11,12-dihidroxy-5Z,8Z,14Z-eicosatrienoic acid	0.4 - 16
11	14(15)-DiHET	14,15-dihidroxy-5Z,8Z,11Z-eicosatrienoic acid	0.4 - 16
12	RvD1	resolvin D1	0.2 - 8
13	LPC 18:0	lysophosphatidylcholine 18:0/0:0	50 - 3200
14	PC 34:1	phosphatidylcholine 16:0/18:1	175 - 11200
15	PE 34:1	phosphatidylethanolamine 16:0/18:1	25 - 1600
16	PG 34:1	phosphatidylglycerol 16:0/18:1	1.25 - 80
17	PI 34:1	phosphatidylinositol 16:0/18:1	12.5 - 800
18	PS 34:1	phosphatidylserine 16:0/18:1	5 - 320
19	PA 34:1	phosphatidic acid 16:0/18:1	25 - 1600
20	LPA 16:0	lysophosphatidic acid 16:0/0:0	1 - 64
21	SM 36:1	sphingomyelin SM d18:1/18:0	125 - 8000
22	SPH 18	sphingosine d18:1	22.5 - 1440
23	S1P	sphingosine-1-phosphate d18:1	10 - 640
24	LA	linoleic acid; FA 18:2	250 - 16000
25	EPA	eicosapentaenoic acid; FA 20:5n-3	6.25 - 400
26	DHA	docosahexaenoic acid; FA 22:6n-3	50 – 3200

Table S2. Internal standards (ISTDs) used for the quantification of 35 plasma lipid species by LC-MRM

ISTD	target concentration [pmol/mL]
AA-d8	5120
TxB2-d4	5.34
5(S)-HETE-d8	12.48
12(S)-HETE-d8	12.48
20-HETE-d6	6.24
11(12)-DiHET-d11	11.44
RvD1-d5	5.24
LPC 17:0_0:0	98.1
LPA 17:0_0:0	58.9
PC 17:0/14:1	104.5
PE 17:0/14:1	111.0
PG 17:0/14:1	70.7
PI 17:0/14:1	62.9
PS 17:0/14:1	67.8
PA 17:0/14:1	118.5
SM d18:1/12:0	77.3
SPH d17:1	350.3
S1P d17:1	273.6
LA-d4	1757
EPA-d5	162.6
DHA-d5	149.9

Table S3. Measured plasma lipid species (absolute quantification)

	Abbreviation	lipid species	mean conc.
1	AA	arachidonic acid; 5Z,8Z,11Z,14Z-eicosatetraenoic acid; FA 20:4	2.86 nmol/mL
2	TxB2	thromboxane B2; FA 20:3;O4	0.168 pmol/mL
3	5(S)-HETE	5S-hidroxyeicosatetraenoic acid; FA 20:4;O	0.304 pmol/mL
4	8(S)-HETE	8S-hidroxyeicosatetraenoic acid; FA 20:4;O	0.151 pmol/mL
5	12(S)-HETE	12S-hidroxyeicosatetraenoic acid; FA 20:4;O	0.514 pmol/mL
6	15(S)-HETE	15S-hidroxyeicosatetraenoic acid; FA 20:4;O	0.134 pmol/mL
7	20-HETE	20-hidroxyeicosatetraenoic acid FA 20:4;O	0.784 pmol/mL
8	5(6)-DiHET	5,6-dihidroxy-8Z,11Z,14Z-eicosatrienoic acid; FA 20:3;O2	0.112 pmol/mL
9	8(9)-DiHET	8,9-dihidroxy-5Z,11Z,14Z-eicosatrienoic acid; FA 20:3;O2	0.112 pmol/mL
10	11(12)-DiHET	11,12-dihidroxy-5Z,8Z,14Z-eicosatrienoic acid; FA 20:3;O2	0.334 pmol/mL
11	14(15)-DiHET	14,15-dihidroxy-5Z,8Z,11Z-eicosatrienoic acid; FA 20:3;O2	0.388 pmol/mL
12	RvD1	resolvin D1; FA 22:6;O3	29.45 fmol/mL
13	LPC 18:0	18:0 monoacylglycerophosphocholine; 18:0 lyso-glycerophosphocholine	17.25 nmol/mL
14	LPC 20:4	20:4 monoacylglycerophosphocholine; 20:4 lyso-glycerophosphocholine	0.774 nmol/mL
15	PC 34:1 (16:0_18:1)	34:1 diacylglycerophosphocholine	127.44 nmol/mL
16	PC 38:4 (18:0_20:4)	38:4 diacylglycerophosphocholine	60.12 nmol/mL
17	PC 38:6 (18:2_20:4)	38:6 diacylglycerophosphocholine	30.729 nmol/mL
18	PC 40:4 (20:0_20:4)	40:4 diacylglycerophosphocholine	1.602 nmol/mL
19	PC 40:6 (20:2_20:4)	40:6 diacylglycerophosphocholine	9.044 nmol/mL
20	PE 34:1 (16:0_18:1)	34:1 diacylglycerophosphoethanolamine	1.566 nmol/mL
21	PE 38:4 (18:0_20:4)	38:4 diacylglycerophosphoethanolamine	0.205 nmol/mL
22	PE 38:6 (18:2_20:4)	38:6 diacylglycerophosphoethanolamine	0.093 nmol/mL
23	PG 34:1 (16:0_18:1)	34:1 diacylglycerophosphoglycerol	55.53 pmol/mL
24	LPI 20:4	20:4 monoacylglycerophosphoinositol; 20:4 lyso-glycerophosphoinositol	1.028 nmol/mL
25	PI 34:1 (16:0_18:1)	34:1 diacylglycerophosphoinositol	2.196 nmol/mL
26	PI 36:4 (16:0_20:4)	36:4 diacylglycerophosphoinositol	3.008 nmol/mL
27	PI 38:4 (18:0_20:4)	38:4 diacylglycerophosphoinositol	19.014 nmol/mL
28	PS 38:4 (18:0_20:4)	38:4 diacylglycerophosphoserine	117.63 pmol/mL
29	SM 34:1 (d18:1/16:0)	N-(hexadecanoyl)-sphing-4-enine-1-phosphocholine; SM 34:1;O2	37.27 nmol/mL
30	SM 36:1 (d18:1/18:0)	N-(octadecanoyl)-sphing-4-enine-1-phosphocholine; SM 36:1;O2	8.59 nmol/mL
31	SM 42:1 (d18:1/24:0)	N-(tetracosanoyl)-sphing-4-enine-1-phosphocholine; SM 42:1;O2	5.37 nmol/mL
32	S1P (d18:1)	sphingosine-1-phosphate; sphing-4-enine-1-phosphate; SPBP 18:1;O2	439.6 pmol/mL
33	LA	linoleic acid; 9Z,12Z-octadecadienoic acid; FA 18:2	69.49 nmol/mL
34	EPA	5Z,8Z,11Z,14Z,17Z-eicosapentaenoic acid; FA 20:5	186.3 pmol/mL
35	DHA	4Z,7Z,10Z,13Z,16Z,19Z-docosahexaenoic acid; FA 22:6	2.124 nmol/mL

Lipid species were measured in plasma samples of diffuse large B-cell lymphoma (DLBCL) patients and controls individuals (CTRL). Mean concentration (mean conc.) for all analytes was calculated (17 DLBCL + 21CTRL). Abbreviated lipid names are shown in red.

Table S4. Between-groups comparison of plasma lipid species: diffuse large B-cell lymphoma (DLBCL) female patients vs. healthy control females (CTRL). Thirty-five plasma lipid species were measured in DLBCL cohort (n=17) and CTRL cohort (n=21). Statistical tests used for the between-group comparisons are shown. p-values were two-tailed: ****p< 0.0001, ***p = 0.0001 to 0.001, **p = 0.001 to 0.01, *p = 0.01-0.05; ns, non-significant p ≥0.05. Fold change (FC) for each variable was calculated by dividing the mean concentration in the DLBCL group by the mean concentration in the CTRL group. Mean analyte concentrations in plasma of DLBCL patients are roughly equal (=), increased (↑) or decreased (↓), compared to CTRL.

Lipid species	Test	p-value	Sig.	FC	Trend
AA	Welch's t-test	0.4683	ns	1.13	↑
TxB2	Mann-Whitney	0.0670	ns	3.12	↑↑↑
5(S)-HETE	Welch's t-test	0.0889	ns	1.30	↑
8(S)-HETE	Welch's t-test on log_{10}-transformed data	0.4210	ns	1.15	↑
12(S)-HETE	Mann-Whitney	0.0031	**	3.15	↑↑↑
15(S)-HETE	Welch's t-test on log_{10}-transformed data	0.0235	*	1.45	↑
20-HETE	Student's t-test on log_{10}-transformed data	0.0189	*	1.45	↑
5(6)-DiHET	Welch's t-test	0.0394	*	1.37	↑
8(9)-DiHET	Welch's t-test	0.4084	ns	1.12	↑
11(12)-DiHET	Welch's t-test on log_{10}-transformed data	0.7199	ns	1.01	=
14(15)-DiHET	Student's t-test on log_{10}-transformed data	0.7707	ns	1.06	↑
RvD1	Welch's t-test	0.0481	*	1.34	↑
LPC 18:0	Student's t-test	0.0920	ns	0.91	↓
LPC 20:4	Student's t-test	0.3861	ns	0.94	↓
PC 34:1	Mann-Whitney	0.6013	ns	1.00	=
PC 38:4	Welch's t-test	0.8317	ns	0.98	=
PC38:6	Mann-Whitney	0.1790	ns	1.13	↑
PC 40:4	Student's t-test	0.1455	ns	1.15	↑
PC 40:6	Student's t-test on log_{10}-transformed data	0.2031	ns	1.15	↑
PE 34:1	Student's t-test	0.5652	ns	1.09	↑
PE 38:4	Welch's t-test	0.2163	ns	1.19	↑
PE 38:6	Student's t-test	0.0709	ns	1.34	↑
PG 34:1	Student's t-test	0.4384	ns	1.10	↑
LPI 20:4	Welch's t-test	0.0077	**	1.51	↑
PI 34:1	Student's t-test	0.0015	**	0.62	↓
PI 36:4	Student's t-test	0.9703	ns	1.00	=
PI 38:4	Student's t-test	0.8501	ns	0.98	=
PS 38:4	Mann-Whitney	0.0079	**	1.49	↑
SM 34:1	Welch's t-test	0.0004	***	1.77	↑
SM 36:1	Welch's t-test on log_{10}-transformed data	0.00008	****	2.10	↑↑
SM 42:1	Welch's t-test	0.0589	ns	1.25	↑

S1P	Mann-Whitney	0.000002	****	1.85	↑
LA	Mann-Whitney	0.0057	**	1.46	↑
EPA	Student's t-test on log_{10}-transformed data	0.1939	ns	1.46	↑
DHA	Student's t-test on log_{10}-transformed data	0.1574	ns	1.44	↑

Table S5. Homogeneity of variance (heteroscedasticity) across DLBCL and control plasma samples

Analyte	Test	p-value	Sig.
S1P	Brown-Forsythe's test	0.00000092	****
SM 34:1	Bartlett's test	0.000055	****
5(S)-HETE	Bartlett's test	0.0005	***
5(6)-DiHET	Bartlett's test	0.0012	**
15(S)-HETE	Bartlett's test (log-transformed data)	0.0013	**
LPI 20:4	Bartlett's test	0.0045	**
SM 36:1	Bartlett's test (log-transformed data)	0.009	**
PC 38:4	Bartlett's test	0.0104	*
8(S)-HETE	Bartlett's test (log-transformed data)	0.0143	*
LA	Brown-Forsythe's test	0.016	*
8(9)-DiHET	Bartlett's test	0.0223	*
11(12)-DiHET	Bartlett's test (log-transformed data)	0.0288	*
PS 38:4	Brown-Forsythe's test	0.0306	*
AA	Bartlett's test	0.0338	*
SM 42:1	Bartlett's test	0.0366	*
PE 38:4	Bartlett's test	0.0398	*

Only heteroscedastic lipid variables are shown

Table S6. The summary of the principal component analysis (PCA) of the lipid dataset obtained through the targeted lipidomics (38 x 35)

principal component	PC1	PC2	PC3	PC4	PC5
Eigenvalue	7.244	5.236	4.592	3.268	2.582
proportion of variance (%)	20.70	14.96	13.12	9.34	7.38
cumulative proportion of variance (%)	20.70	35.66	48.78	58.12	65.50

Table S7. **Spearman correlations among measured plasma lipid species in DLBCL and CTRL cohorts. ϱ is a Spearman's correlation coefficient, q is a BH-corrected p-value at a false discovery rate (FDR) of 5%; the significance of the q-value is shown (Sig.). Strong inter-lipid correlations in the DLBCL group that were moderate or non-significant in the CTRL are in italics.**

DLBCL group

Lipid_1	Lipid_2	P	p-value	q-value	Sig.
PC 38:6	PC 40:6	0.931	5.6×10^{-8}	3.4×10^{-5}	****
11(12)-DiHET	14(15)-DiHET	0.907	5.2×10^{-7}	0.00015	***
AA	5(S)-HETE	0.886	2.2×10^{-6}	0.00044	***
PI 34:1	PI 36:4	0.878	3.7×10^{-6}	0.00055	***
EPA	DHA	0.841	2.4×10^{-5}	0.0028	**
SM 34:1	SM 36:1	0.836	2.9×10^{-5}	0.0029	**
AA	*8(S)-HETE*	0.828	0.00004	0.0034	**
LA	*DHA*	0.782	0.00021	0.0156	*
5(S)-HETE	*8(S)-HETE*	0.777	0.00025	0.0162	*
SM 36:1	SM 42:1	0.762	0.00037	0.0223	*
AA	*EPA*	0.752	0.00049	0.0266	*
PC 38:4	*PC 40:6*	0.740	0.00068	0.0337	*
11(12)-DiHET	*DHA*	0.728	0.00092	0.0423	*
PC 40:6	*PE 38:6*	0.719	0.0012	0.0491	*

CTRL group

Lipid_1	Lipid_2	P	p-value	q-value	Sig.
11(12)-DiHET	14(15)-DiHET	0.933	7.3×10^{-10}	4.4×10^{-7}	****
PE 38:4	PG 34:1	0.918	4.5×10^{-9}	1.3×10^{-6}	****
PC 38:6	PC 40:6	0.912	9×10^{-9}	1.8×10^{-6}	****
8(S)-HETE	15(S)-HETE	0.881	1.3×10^{-7}	0.00002	****
PI 36:4	PI 38:4	0.851	1×10^{-6}	0.00012	***
SM 34:1	SM 42:1	0.847	1.3×10^{-6}	0.00012	***
SM 34:1	SM 36:1	0.846	1.4×10^{-6}	0.00012	***
PE 34:1	PG 34:1	0.829	3.5×10^{-6}	0.00026	***
PC 38:6	DHA	0.812	7.9×10^{-6}	0.00052	***
EPA	DHA	0.797	1.5×10^{-5}	0.00085	***
AA	5(S)-HETE	0.796	1.6×10^{-5}	0.00085	***
PC 40:6	DHA	0.793	1.8×10^{-5}	0.00089	***
PI 34:1	PI 36:4	0.760	6.5×10^{-5}	0.0030	**
PE 34:1	PE 38:4	0.748	9.7×10^{-5}	0.0041	**
8(9)-DiHET	14(15)-DiHET	0.702	0.00039	0.0154	*
AA	S1P	−0.700	0.00043	0.0158	*
5(S)-HETE	LA	0.693	0.0005	0.0175	*
PI 34:1	PI 38:4	0.686	0.0006	0.0199	*
AA	LA	0.672	0.00086	0.0256	*
SM 36:1	SM 42:1	0.671	0.00086	0.0256	*
PC 40:6	EPA	0.665	0.001	0.0285	*
8(9)-DiHET	11(12)-DiHET	0.659	0.0012	0.0298	*
S1P	LA	−0.660	0.0012	0.0298	*
12(S)-HETE	PI 38:4	0.657	0.0012	0.0298	*
5(S)-HETE	8(S)-HETE	0.649	0.0015	0.0346	*
12(S)-HETE	PI 36:4	0.637	0.0019	0.0433	*
8(S)-HETE	8(9)-DiHET	0.634	0.002	0.0433	*
PC 38:6	EPA	0.634	0.002	0.0433	*
5(6)-DiHET	11(12)-DiHET	0.629	0.0023	0.0433	*

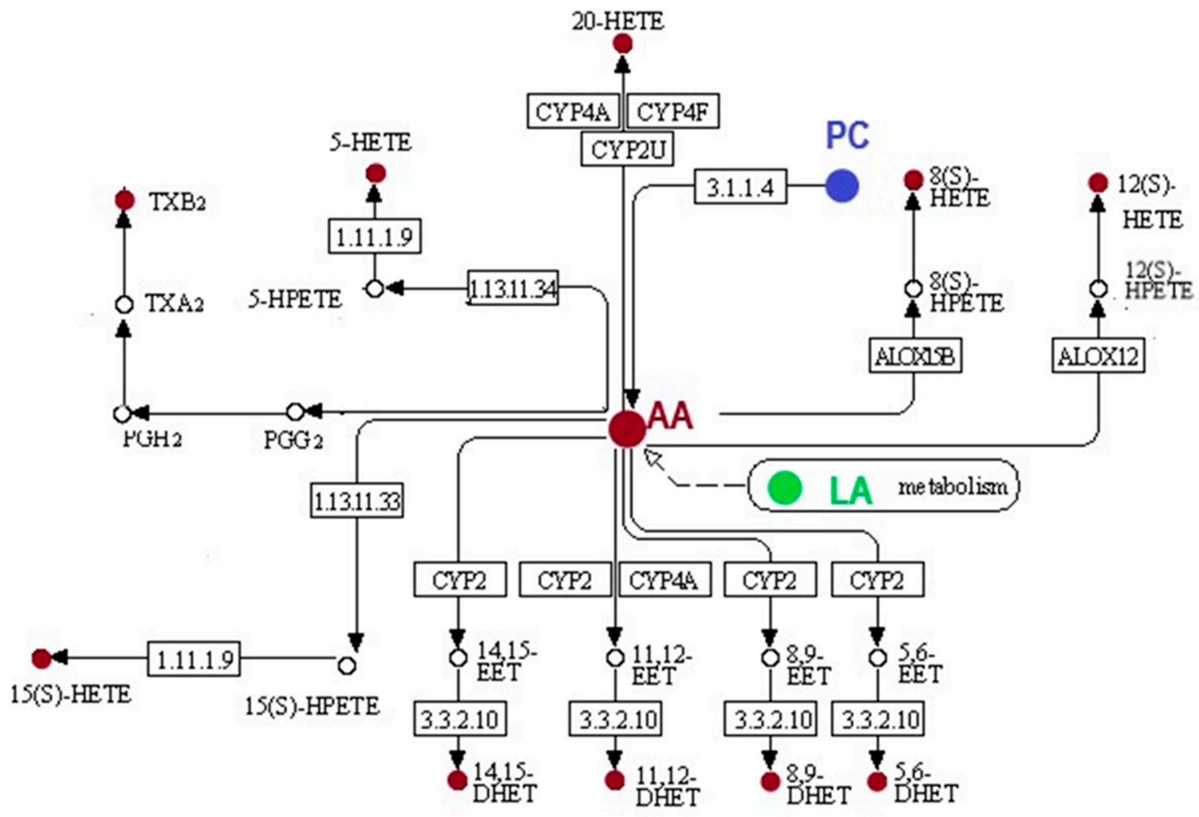

Figure S1. Simplified metabolic pathway of arachidonic acid (AA). The figure shows mainly the measured eicosanoids and their metabolic precursors. Phosphatidylcholine (PC) from the plasma membrane is a precursor of AA, and linoleic acid (LA) entry is also displayed. The map was downloaded from https://www.genome.jp/pathway/map00590 and simplified.

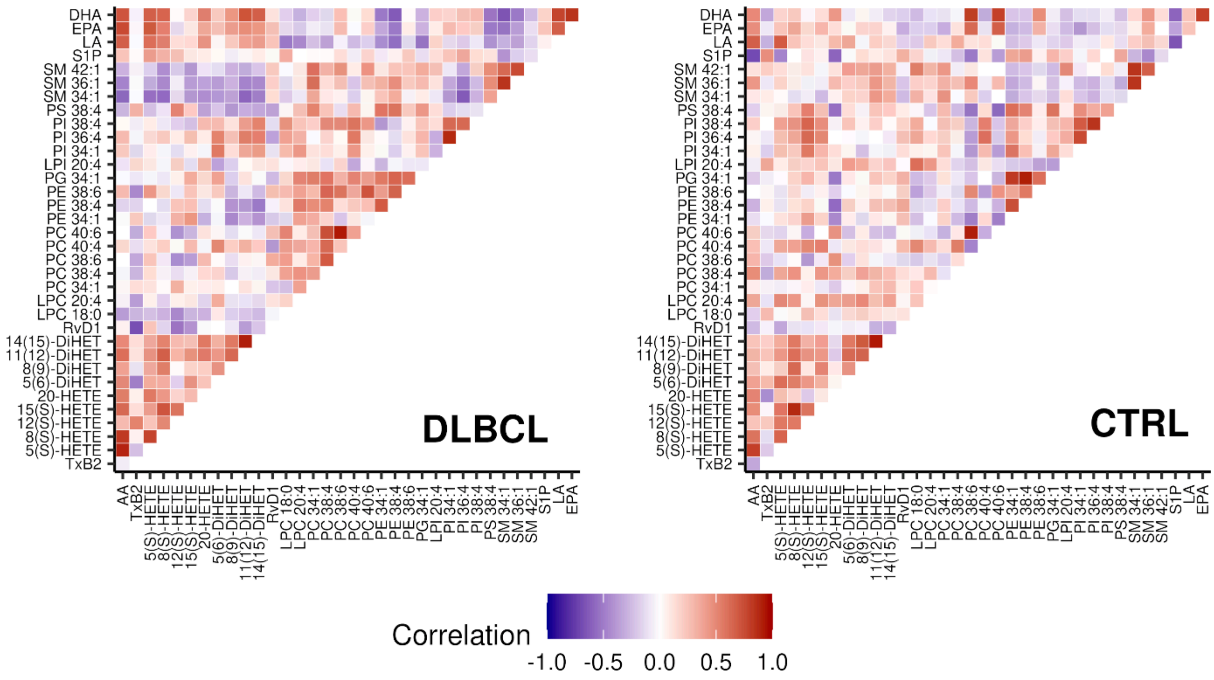

Figure S2. Heatmaps by Spearman correlation coefficients between pairs of lipid species in DLBCL and CTRL group. The rows and columns represent the lipid species. The magnitude of correlation is proportional to the colour intensity.
